# A new Caribbean species of *Hylaeanura* Arlé, 1966 (Collembola, Neanuridae, Pseudachorutinae)

**DOI:** 10.3897/zookeys.961.47227

**Published:** 2020-08-19

**Authors:** Claudia M. Ospina-Sánchez, José G. Palacios-Vargas, Grizelle González

**Affiliations:** 1 USDA-FS, International Institute of Tropical Forestry, Río Piedras, 00926-1119, Puerto Rico International Institute of Tropical Forestry Río Piedras Puerto Rico; 2 Laboratorio de Ecología y Sistemática de Microartrópodos, Departamento de Ecología y Recursos Naturales, Facultad de Ciencias, Universidad Nacional Autónoma de México, 04510 México, D.F., México Universidad Nacional Autónoma de México México Mexico

**Keywords:** Island, Luquillo Experimental Forest, Puerto Rico, subtropical forest, taxonomy

## Abstract

We here describe a new Collembola species, *Hylaeanuraemiliae***sp. nov.**, from the Luquillo Experimental Forest in Puerto Rico. We describe *H.emiliae***sp. nov.** as a distinct species based on the enlarged sensilla s3 in antennal segment IV, the absence of modified sensorial setae in abdominal segment IV and the presence of four setae on each dens. An updated key with illustrations for the identification of worldwide species of the genus is included.

## Introduction

In Puerto Rico, most studies of arthropod community dynamics have been performed in the Luquillo Mountains (Richardson 1999; González and Barberena 2017; Quiñones et al. 2018). The Luquillo Experimental Forest (LEF) contains four forest types defined by elevation and distinct tree species composition (Gould et al. 2006; Weaver and Gould 2013). The present study focuses on three of them: (1) The Tabonuco (*Dacryodesexcelsa* Vahl) forest occupies areas below 600 m, (2) the mid-elevation, Palo Colorado (*Cyrillaracemiflora* L.) forest occurs in areas above the cloud condensation level from 600–900 m, and (3) the Elfin forest (*Tabebuiarigida* Urban), with stunted vegetation and waterlogged anoxic soils, is located only on the highest peaks above 900 m (Gould et al. 2006). These forests represent the subtropical wet and subtropical rain forest life zones in Puerto Rico (Ewel and Whitmore 1973).

In most studies of litter and soil fauna in the LEF, Collembola are an important group for ecosystem functioning because of their dominant abundance and their key responses to changes in disturbance, altitude and vegetation type (Schowalter and Ganio 1999; Schowalter et al. 2003; Richardson et al. 2005; Richardson et al. 2010; Schowalter et al. 2014). In Puerto Rico, collembolans are well known in comparison to other groups of soil arthropods. However, not all Collembola species from LEF have been identified (González and Barberena 2017). In a recent survey made between 2014 and 2015, 16 families 37 genera and 60 species/morphospecies were identified, and among these, 15 are new species to science (Ospina-Sánchez 2019). The purpose of this paper is to describe a new species of *Hylaeanura* Arlé, 1966.

The genus *Hylaeanura* was conceived by Arlé (1966) to place *Paranurellainfima* described by himself in 1959. So far only four species are known: *Hylaeanuranepalensis* (Yosii, 1966), originally described as *Paranuranepalensis* from the Himalayas; *H.nohbecana* Vázquez, Cutz-Pool & Palacios-Vargas, 1998, from Mexico; *H.mendoncae* Zeppelini & Palacios-Vargas, 2013, from Brazil and *H.infima* from Brazil, French Guiana and Peru (Najt et al. 1990). The first taxonomic report of the genus from Puerto Rico was *H.infima* (Ospina-Sánchez et al. 2020) and it now includes the new species *Hylaeanuraemiliae*.

## Materials and methods

The material used to describe the new *Hylaeanura* species came from the survey of the Collembola microhabitats in the Luquillo Mountains conducted in 2014 and 2015 along three forest types (Tabonuco, *Dacryodesexcelsa*; Palo Colorado, *Cyrillaracemiflora* and Elfin forest, *Tabebuiarigida*). Collembola were extracted from soil and litter samples using a Berlese-Tullgren funnel and stored in 95% ethanol. They were cleared using Nesbitt solution and fixed in slides using Mac André II solution (Mari Mutt 1976). To harden the solution, the slides were dried in a slide warmer at 45 to 50 °C for seven days. Finally, each specimen was labeled with its collecting data. Specimens were examined with a Leica DM500 phase-contrast microscope. The drawings were made with the aid of a drawing tube.

**Abbreviations**:

**a.s.l.** above sea level

**Abd** abdominal segment

**Ant** antennal segment

**PAO** Postantennal Organ

**S** sensilla

**Sgd** dorsal guard sensillum of Ant III

**Sgv** ventral guard sensillum of Ant III

**ss** sensorial setae

**Tita** tibiotarsi

**Th** thoracic segment

## Taxonomy

### Poduromorpha

**Neanuridae**

**Pseudachorutinae**

#### Hylaeanura

Taxon classificationAnimaliaCollembolaNeanuridae

Arlé, 1966

D4657C2D-8A0E-5A1F-A74E-C4209996E2AA

##### Diagnosis

(modified from Zeppelini and Palacios-Vargas 2013). Habitus of *Paranurella* or *Kenyura*, i.e., reduced appendices and without pigment; less than 1.0 mm in size; without eyes or at most 2 eyes per side; antennae shorter than half the cephalic diagonal, Ant. IV with 7 sensilla, S8 hypertrophied; mandible with one to three teeth, maxilla styliform; legs very short, ungues without teeth and unguiculus, tenent hairs not developed. Ventral tube with 3+3 setae; tenaculum 2+2 to 3+3, furcula very reduced, each dens with 3 setae, mucro minute or lacking. Body chaetotaxy reduced and with very small setae.

#### Hylaeanura emiliae sp. nov.

Taxon classificationAnimaliaCollembolaNeanuridae

7E2F25BC-F0ED-500F-8259-A42C3BB45E43

http://zoobank.org/7361B653-81A6-4781-9F58-9CF8AAA05CCE

Figures 1–3 , 4–9 , Table 1

##### Type material.

***Holotype.*** Female on slide. ***Paratypes***: 1 female and 1 juvenile on slide. All the type material kept at corresponding author’s institution.

##### Type locality.

Puerto Rico, Luquillo, Luquillo Mountains, Toro Trail 1, 18°16'40"N, 65°50'53"W; 815 m a.s.l.; ex soil, *Cyrillaracemiflora* forest type, 18 November 2014. All specimens were extracted using Berlese-Tullgren funnels from samples collected in leaf litter and soil at the Luquillo Mountains. C. M. Ospina and M. Rivera leg.

##### Other material.

Female on slide, Puerto Rico, Luquillo, Luquillo Mountains, Toro Trail 2, 18°16'40.3"N, 65°51'01"W, *Cyrillaracemiflora* forest type, leaf litter, 795 m a.s.l., 18 November 2014, C.M.Ospina leg. One (1) juvenile on slide, Puerto Rico, Luquillo, Luquillo Mountains, Toro Trail 1, 18°16'40"N, 65°50'53"W, *Cyrillaracemiflora* forest type, leaf litter, 815 m a.s.l., 18 February 2015, C. M. Ospina leg.

##### Diagnosis.

*Hylaeanuraemiliae* sp. nov. has an enlarged sensillas S3 and S8 on Ant IV, Abd IV without modified setae, and four setae on each dens.

##### Description.

**Figures 1–3.**
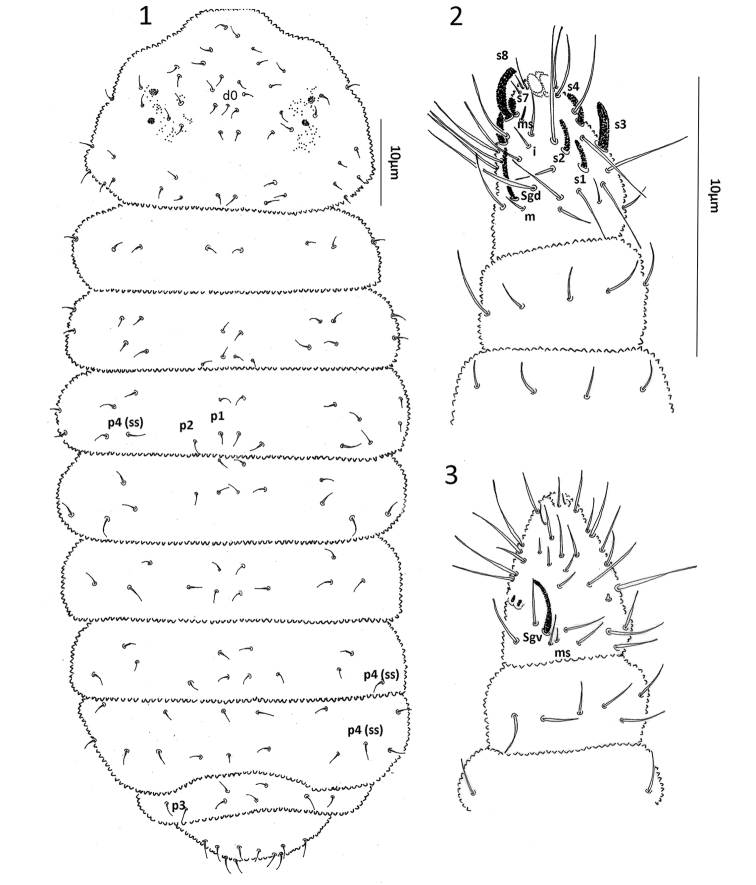
*Hylaeanuraemiliae* sp. nov. **1** Dorsal chaetotaxy **2**Ant. I–IV dorsal view **3**Ant. I–IV ventral view.

Length of the holotype 1050 µm (female paratype 653 µm and juvenile paratype 550 µm). Specimens in ethanol without color, body with coarse granulation and without tubercles. Body setae short and smooth, the sensorial setae longer than ordinary setae, both acuminate (Fig. 1).

**Figures 4–9.**
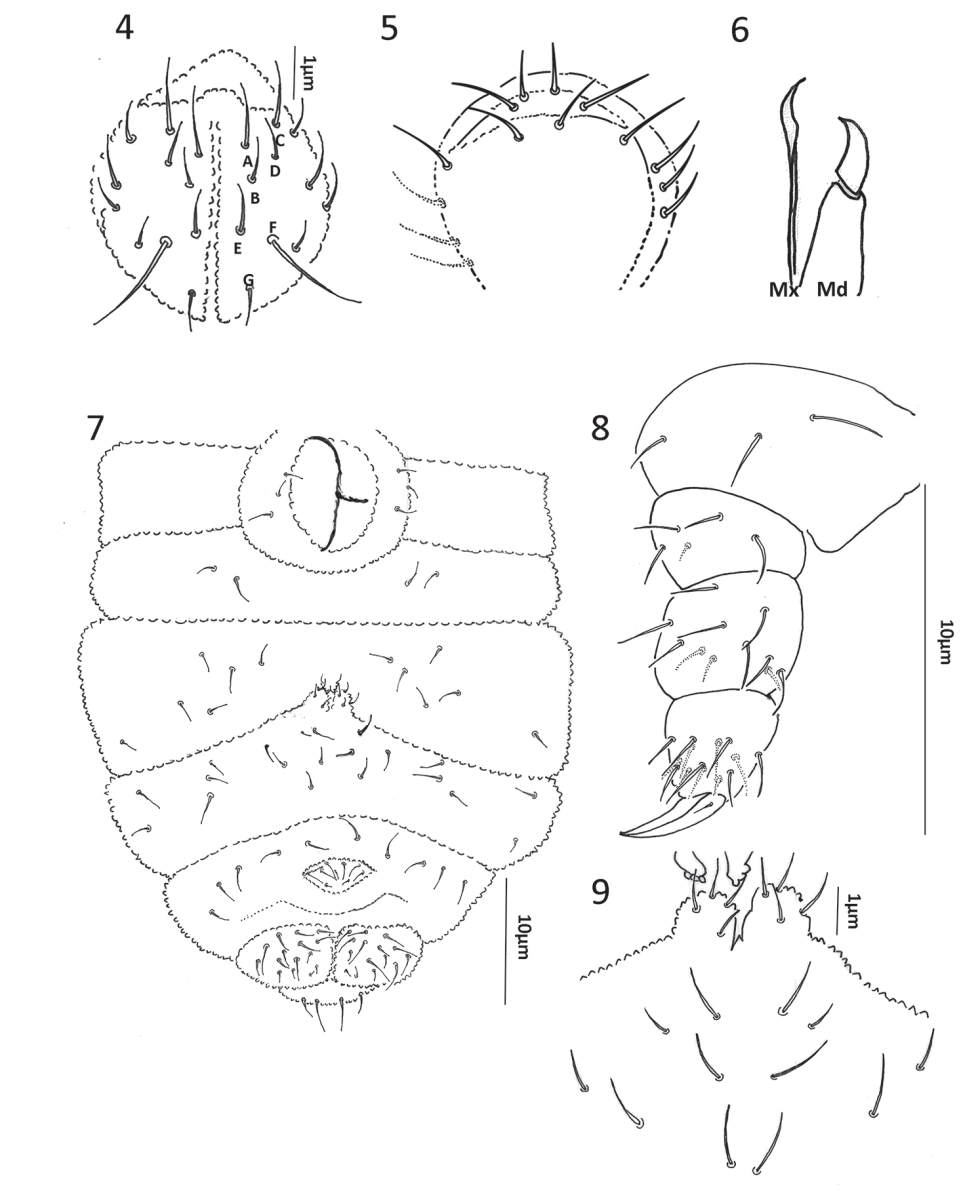
*Hylaeanuraemiliae* sp. nov. **4** Labium **5** Labrum **6** Mandible (Md) and Maxillae (Mx) **7** Ventral abdominal chaetotaxy **8** leg I **9** reduced furcula and tenaculum.

Head: Antenna smaller (0.47×) than cephalic diagonal. Ant III and IV fused dorsally. Ant IV dorsally with a trilobed apical vesicle; subapical organite absent and dorsoexternal microsensillum, 7 subcylindrical sensilla, S8 hypertrophied, S7 smaller and S3 large; 12 long setae plus i (Fig. 2); Ant III sense organ with two small internal straight sensilla and two subequal subcylindrical guard sensilla; ventral microsensillum present (Fig. 3); Ant II with 11 setae; Ant I with six setae. Eyes 2+2 in a pigmented patch. PAO absent. Head dorsal quetotaxy as in Fig. 1, unpaired setae d0 present. Labium with a total of 11 setae per side with setae A to G, setae C and D slightly displaced apically (Fig. 4). Labral chaetotaxy 4, 2, 2 (Fig. 5). Mandible with one tooth, maxilla styliform (Fig. 6).

Body: Ordinary body setae smooth, distributed as in Fig. 1. Th I with 3+3 setae. Sensory setae (ss) similar to body setae, in position p4 in all segments and distributed on Th. II-Abd V as 11/11111. Ventral chaetotaxy as in Fig. 7. Female genital plate with 3+3 pregenital setae, 7 circumgenital and 2 eugenital (Fig. 7). Male genital plate not seen. Each anal ventral lobe with 14 setae.

Legs: Chaetotaxy of legs I–III as follow: Coxae with 3, 5, 7 trochanter with 5, 5, 4; femora 11, 11, 10 and tita with 16, 16, 14 setae (Fig. 8). Tenent hair not developed and unguiculus absent; claws without teeth.

Collophore with 3+3 setae; tenaculum with 3+3 teeth and without setae; furcula reduced, manubrium reduce with 6+6 setae in Abd III ventrally; dens with four setae, mucro absent (Fig. 9).

##### Etymology.

This species is dedicated to the daughter of the senior author, Emilia, who was born while this research was being conducted.

##### Distribution.

*Hylaeanuraemiliae* sp. nov. is only known from the Luquillo Mountains in the *Cyrillaracemiflora* forest type, on the Toro Trail between 795–815 m a.s.l.

##### Fenology.

*Hylaeanuraemiliae* sp. nov. was extracted from leaf litter and soil in both dry and rainy seasons during November 2014 and May and August 2015.

### Identification key to the species of *Hylaeanura* Arlé, 1966

(updated from Zeppelini and Palacios-Vargas 2013)

**Table:** 

1	Without eyes	**2**
–	With 2 eyes per side	**3**
2	Sensorial seta on Abd IV candle-flame shaped. Dens with a minute mucro present	***H.nohbecana* Vázquez, Cutz-Pool & Palacios-Vargas, 1998**
–	Sensorial seta on Abd IV sensillum shaped. Dens without mucro	***H.infima* (Arlé, 1959)**
3	Dorsal and ventral guard sensilla sinuous (Sensorial organ Ant Ill). Mandible with two teeth	***H.mendoncae* Zeppelini & Palacios-Vargas, 2013**
–	Dorsal and ventral guard sensilla straight, Mandible with 1 or 3 teeth	**4**
4	Tenaculum with 2+2 teeth. Mandible with 3 teeth	***H.nepalensis* (Yosii, 1966)**
–	Tenaculum with 3+3 teeth. Mandible with 1 tooth	***H.emiliae* sp. nov.**

## Discussion

The description of *Hylaeanuraemiliae* sp. nov. fits with the most recent genus diagnosis proposed by Zeppelini and Palacios-Vargas (2013). Although in the diagnosis of the genus, the dens each have three setae and *H.emiliae* sp. nov. has four setae in each, the number of setae on the dens, as well as the shape of the mucro, are variable characters among the species (D’Haese 2003). The similar genus *Kenyura* includes species having numerous teeth on the mandible, S8 is normal and the presence of pigmentation (Arlé 1966, Vázquez et al. 1998).

*Hylaeanuraemiliae* sp. nov. is different from other *Hylaeanura* due to the enlargement of the sensilla s3 in Ant IV, the absence of modified S setae in Abd IV and the presence of four setae in the dens. Additionally, it has an unpaired seta d0 in the head. This setae is absent in other described species of the genus, but defines a unique difference within head chaetotaxy.

Using the comparative morphology of *Hylaeanura* presented by Zeppelini and Palacios-Vargas (2013), the character combination of the new species is different from all others previously described. According to their descriptions the most similar is *Hylaeanuranepalensis*, but it differs in size, the presence of 2+2 tenacular teeth and three mandibular teeth (Yosii 1966).

*Hylaeanurainfima* is the smallest species and has a sensillum of Abd IV of setae shape. It differs from the new species by the absence of eyes and the presence of two teeth in the mandible and the presence of three setae in dens (Arlé 1966, Thibaud and Massoud 1983).

*Hylaeanuranohbecana* is the biggest *Hylaeanura*, and is similar to the new species in the straight shape of the guard sensillum of Ant IV, the differences appearing in the chaetotaxy: the absence of unpaired setae d0 in the head, the presence of setae a3 in Abd II-IV and the position of the ss in p3 in Abd I. Additionally, *H.nohbecana* has no eyes, the furcula has two small dens each bearing 3 setae and a small vestigial mucro (Vázquez et al. 1998).

The most recently described species, *H.mendoncae* differs from *H.emiliae* sp. nov. in the position of ss in Abd I to III in p3. In Abd IV, ss is also in position p3 but in the shape of a candle-flame setae. In Abd V ss is in position p2. The furcula is reduced and dens have 3 setae each without a mucro (Zeppelini and Palacios-Vargas 2013). Similar to *H.emiliae* sp. nov., *H.mendoncae* has 6 + 6 setae present ventrally in Abd III as vestigial manubrium. The differences between described *Hylaeanura* species are described in Table 1.

**Table 1.** Summary of main characters of described species of *Hylaeanura* Arlé, 1966.

Species	Total length (µ)	Ventral guard sensillum	Dorsal guard sensillum	Mandible teeth	Eyes per side of head	Shape Abd IV sensillum	Tenaculum teeth	Setae in dens	Setae in manubrium	Mucro
*H.infima*	500	st	st	2	0	ss	?	3	2	–
*H.nohbecana*	1000	st	st	3	0	cf	3+3	3	2	+
*H.nepalensis*	700	st	st	3	2	ss	2+2	3	2	–
*H.mendoncae*	600	si	si	2	2	ss	2+2	3	0 (6)	–
*H.emiliae* sp. nov.	850	st	st	1	2	ss	3+3	4	0 (6)	–

st, straight si, sinuous cf, candle-flame shaped ss, sensillum acuminated ?, no information included in the original description -, absent +, present

## Supplementary Material

XML Treatment for Hylaeanura

XML Treatment for Hylaeanura emiliae
